# #KindnessbyPost: a pre-post, observational evaluation and feasibility study of a participatory public mental health intervention

**DOI:** 10.1186/s12889-026-28170-x

**Published:** 2026-07-22

**Authors:** Marie Le Novere, Caroline S. Clarke, Rebecca Jones, Katey Warran, Brynmor Lloyd-Evans, Luke Sheridan Rains

**Affiliations:** 1 UCL Research Department of Primary Care and Population Health, University College London, London, UK; 2 Division of Psychiatry, University College London, London, UK; 3 School of Health in Social Science, University of Edinburgh, Edinburgh, UK; 4 WHO Collaborating Centre for Arts and Health, Social Biobehavioural Research Group, University College London, London, UK

**Keywords:** Social Isolation, Loneliness, Wellbeing, Evaluation, Health Economics, Acts of Kindness

## Abstract

**Background:**

#KindnessbyPost (KbP) is a “national kindness exchange”. Participants send and receive a card by post to different, unknown people containing messages of good will. Previous evidence suggests participation in KbP may reduce loneliness and improve wellbeing. We evaluated the outcomes and administrative costs of KbP and explored the feasibility of collecting health economic measures for future research.

**Methods:**

We used a pre-post, observational study design. Participants in two separate KbP exchanges in 2021 were invited to complete an online questionnaire. It included self-reported measures quantifying psychological wellbeing and loneliness, capability, and healthcare service use. Data were collected at three time-points per exchange: exchange-start (baseline), exchange-end (2 weeks), and 3 months post-exchange. Administrative cost data were obtained from the KbP programme organisers.

**Results:**

Across both exchanges, 1,285 responses were received from 1,142 participants. 143 participants completed questionnaires at both exchanges. In total, 623 (48%) participants completed follow-ups at exchange-end, and 414 (32%) at 3 months post-exchange. 70% (n = 789) of participants were aged between 31 and 60 years. 94% (n = 1,075) of participants were female and 95% (n = 1,078) were of white ethnicity. Compared to baseline, loneliness reduced and wellbeing increased at exchange end (change in loneliness = ‑0.21 units, 95% CI: -0.29 to -0.12; change in wellbeing = 0.68 units, 95% CI: 0.47 to 0.88, respectively) and at 3 months post-exchange (loneliness change = -0.40 units, 95% CI: -0.50 to -0.30; wellbeing change = 1.01 units, 95% CI: 0.77 to 1.25, respectively). Capability scores (mean (SD) were calculated at baseline: 0.74 (0.18); exchange-end: 0.75 (0.19); and 3 months: 0.76 (0.18)) from available responses. The average administrative cost of delivering the intervention was £0.67-£0.87 per participant, not including in-kind contributions.

**Conclusions:**

We observed a small, sustained improvement to people’s wellbeing and loneliness following participation in a KbP exchange. Given the study design, causal attribution to KbP is uncertain. However, our results are consistent with previous evaluations and offer preliminary evidence that KbP may be an effective mental wellbeing intervention. Given its low administrative cost and highly scalable design, KbP could also be a cost-effective intervention for improving public mental health, warranting definitive evaluation of effectiveness and cost-effectiveness in future. Our study also demonstrated the feasibility of collecting wellbeing and health economic outcome measures needed for future research.

**Supplementary Information:**

The online version contains supplementary material available at https://doi.org/10.1186/s12889-026-28170-x.

## Background

Loneliness and social isolation are distinct but related constructs: loneliness is a negative emotional state resulting from a perceived deficiency in the quantity and/or quality of someone’s social relationships [1]; while social isolation refers to a lack of contact or social relationships [2]. In March 2022, data from the Office for National Statistics indicated that 23.6% of adults (estimated to be around 12.3 million people) in the United Kingdom reported feeling lonely at least some of the time [3]. There is growing evidence linking loneliness and social isolation to a wide range of mental and physical health conditions, including risk of premature death from all causes, which may be comparable in risk to smoking, obesity; physical inactivity, as well as cardiovascular disease [4]; depression, anxiety, and suicide [5]. The prevalence of poor wellbeing, social isolation, and loneliness is particularly high amongst those with mental or physical disability, and especially amongst those who are younger, economically inactive, living alone, and who have poor access to green spaces and other community amenities [6]. Lower social support and loneliness are also associated with more severe symptoms, poorer recovery outcomes, and worse social functioning for people with common mental disorders [7]. Early evidence from populations with severe mental illnesses suggest similar associations, including with more severe depression, psychotic symptoms, and anxiety, as well as poorer social support and quality of life [8]. The most studied cohort though is older adults, in whom there is now substantial evidence of high rates of loneliness and social isolation, and of their impact on greater morbidity, shortened life expectancy [9], dementia [10], and amongst those with a history of heart failure, a four-fold increased risk of death [5].

Interventions that improve feelings of social connectedness and reduce loneliness have the potential to produce significant health benefits in the general population, for local communities, and especially amongst those who are most vulnerable [11]. Of note, it has been theorised that leisure engagement (which includes creative and community activities) can support health and wellbeing through underlying social mechanisms, such as reducing loneliness and improving social networks and community cohesion [12]. Examples include Men’s Sheds (community-based organisations that provide meaningful spaces for older men) reducing social isolation to improve wellbeing, group creative writing fostering belonging to support mental health [13, 14], and community singing groups facilitating social cohesion to reduce symptoms of post-natal depression [15]. There is also more and more evidence suggesting that creative arts activities, including painting, drawing and creative writing, are beneficial for general wellbeing and mental health [16, 17].

#KindnessByPost (KbP) is a national “kindness exchange” that has the potential to improve participants’ wellbeing by increasing their sense of connectedness to others and reducing feelings of social isolation and loneliness. [18]. KbP works on a “secret Santa” model: each participant sends a card to and receives one from different, unknown people. Participants can share images of the cards and their reactions to them on social media using the #KindnessbyPost hashtag. KbP is organised as discrete exchanges, and people register to participate in the upcoming exchange. Exchanges were planned around dates when people may feel especially lonely, such as Valentine’s Day. Some targeted the general population (e.g. summer exchange), while others focused more on specific social or cultural groups (e.g. Ramadan exchange). Random acts of kindness can improve the wellbeing of both the giver and receiver [19–23] and, in the case of KbP, have the potential to improve social connectedness and reduce loneliness [24]. Kind acts can make individuals feel more positive about themselves [19] and provide benefits to feelings of wellbeing [25], whilst receiving acts of kindness can make people feel acknowledged and valued by others [26].

KbP is inexpensive to administer and, in principle, highly scalable, giving it the potential to be a public health intervention that could improve wellbeing both in the general population and amongst vulnerable groups. Over 6,000 people participated in the six KbP exchanges prior to this study; and exchanges have grown in popularity. This figure includes 3,500 people who took part in Covid-19 [27] lockdown exchanges, which confirmed the attraction of KbP when ways to tackle isolation without face-to-face contact became more important than previously. Given the rising popularity of KbP, its modest cost to administer, and potential impact, it is important that evidence regarding its effectiveness be explored. An evaluation of KbP 2020 #GreatBritishValentine event offered some preliminary evidence, finding significant improvements in people’s feelings of wellbeing, loneliness, sense of belonging, and hope following the exchange [28]. However, this evaluation had several limitations that we sought to address in the present study. These include that the generalisability of its evidence base was limited by only including data from one exchange, and it did not investigate whether these effects were sustained longer term. In this study, we delivered a more robust evaluation, in which we collected data from two separate exchanges instead of one, investigated whether any changes were sustained 3 months after the end of the exchange, and calculated the administrative costs of KbP from the perspective of the Mental Health Collective.

The aims of this work were firstly to estimate the impact of taking part in a #KindnessByPost exchange on participants’ feelings of wellbeing and loneliness, both at the end of the exchange and 3 months later, which could be used to inform a future study of effectiveness; secondly to estimate the cost of delivering the KbP from the perspective of the Mental Health Collective (MHC); and thirdly to evaluate the feasibility of collecting health economic measures for cost-effectiveness analysis alongside a future larger study.

## Methods

### Ethical approval and informed consent

This study was approved by the UCL Research Ethics Committee (ref: 14,649/004). Those who signed up to take part in a KbP exchange were asked by email if they would volunteer to complete an online questionnaire and were provided with a link. The first page of the questionnaire provided information regarding the study, including contact details for who to contact with questions, as well as details of how their data would be used, and that they could still take part in the exchanges without participating in the present study. They were informed that by completing the questionnaire, they gave their consent for their de-identified data to be included in the study. Data were collected by the MHC, who are responsible for developing and running KbP. They cleaned and anonymised the dataset prior to it being transferred to researchers based in UCL for analysis.

### Participant population

Participants are invited to KbP exchanges via newspapers, radio, MHC mailing lists, and on social media. Anyone living in the United Kingdom and over 13 years old, or younger if in partnership with an adult, can take part. Participants sign up via a dedicated KbP website [29]. An online platform allocates each participant a recipient to whom to send a card or letter. Participants are informed that there is no guarantee that they will receive a card. However, those who do not receive cards first time around may use a ‘back-up system’, where volunteers send cards to people who did not receive one. MHC estimates that around 80% of participants receive cards in the first round, increasing to 97% of participants after employing the back-up system [30]. There were six exchanges in 2020/21. We conducted the questionnaire during two successive exchanges: the February 2021 #GreatBritishValentine (GBV) exchange and the June 2021 summer exchange. All participants over the age of 18 were invited to participate in the present study when they registered online for these exchanges. There were no additional exchanges between February and June 2021.

### Study design

We used a cross-sectional, repeated measures design with convenience sampling. Working with the MHC, the research team developed an online questionnaire using the Google Forms platform that collected data on subjective wellbeing, loneliness and health economics outcomes. It was conducted by the MHC at three time-points per exchange: exchange start (baseline), exchange end (2 weeks), and 3 months post-exchange. Questionnaires were open for 2 weeks for participants to complete. Participants were sent the questionnaire link via email along with reminders. A raffle was held after each questionnaire closed for all participants who had responded at that time point, with a £100 voucher prize for a well-known shop in the UK. A copy of the questionnaires is available in Additional files 1—3. Data collection for the first exchange was completed prior to the commencement of data collection for the second exchange.

Questionnaire items included:

- Demographics, including gender, ethnicity, and age group.
- Wellbeing, measured using the seven-item Short Warwick-Edinburgh Mental Wellbeing Scale (SWEMWBS). Each item is scored with a Likert-scale between 1–5. Overall raw scores range from 7–35, with higher scores indicating more positive wellbeing [31].
- Loneliness, measured using the three-item version of University of California at Los Angeles (UCLA) Loneliness Scale version-3 ULS-3. Each item is scored with a Likert-scale between 1–3. Overall raw scores range from 3–9, with higher scores indicating greater loneliness [32].
- Capability, measured using the ICEpop CAPability measure for Adults (ICECAP-A), which captures information on attachment, stability, achievement, enjoyment and autonomy, scoring each attribute from 1 for no capability to 4 for full capability [33]. Responses can be combined into a ‘capability index score‘ using a published algorithm created via a valuation study, which is standardised on a scale from 0 to 1, where 1 is maximum capability according to the five attributes, and 0 signifies worst or minimum capability.
- Resource use, measured using a reduced Client Service Receipt Inventory (CSRI) questionnaire [34], focusing on items relevant to the standard UK NHS and personal social services (PSS) perspective [35]. We considered use of primary and community health care contacts in the previous three months. This included General Practitioners (GP), Practice Nurse (GP Nurse), Therapists (Speech and language, Physio, and Occupational) and contacts with NHS Direct/111 as well as any talking therapy sessions (one-to-one and/or group). We also asked an open-ended question regarding whether there had been any other changes to the health care support accessed over the last 3 months, in order to assess what other types of health care services should be incorporated in a future larger study, while minimising the numbers of questions on the form.

Additional data for calculating the administrative costs were provided by the MHC detailing the amount of time spent organising a KbP exchange, including staff timesheets and running costs.

Both the SWEMWBS and UCLA loneliness measures have been found to have good internal consistency, convergent and construct validity, and test–retest reliability [32, 36–38].

### Data preparation

Initially, the MHC matched questionnaire responses for unique respondents across data collection time points and exchanges based on email address. Where multiple responses were submitted by the same participant, the most complete set of responses per participant was kept. Where this still gave a choice of sets, the chronologically first were kept. Following this step, there were no missing data from completed questionnaires. Questionnaire data were then combined into a single dataset that linked participants’ responses across different time points and exchanges. Email addresses were replaced with participant IDs to pseudonymise the data before being provided to the UCL research team for analysis.

### Statistical analysis

#### Loneliness and wellbeing

Participant demographics at baseline were summarised using counts and percentages. Loneliness and wellbeing measures were summarised separately for each time point using means and standard deviations (SD). A separate multilevel, hierarchical model was fitted for each of the two outcomes (loneliness and wellbeing) with measures from each of the two card exchanges (GBV and Summer) and each of the three time points (baseline; post-exchange and 3 months post-exchange) as the repeated measures outcome. A random effect of participant was used to account for correlations between repeated measures from individuals at different time points as well as for correlations between scores on the two exchanges for individuals who participated in both. The explanatory variable was the time point at each measurement. Estimates of change in loneliness and wellbeing from baseline to the two follow-up time points were obtained from these statistical models. Standardised scores for both of the primary outcomes at every time point were calculated using the mean and SD of the measure at baseline. Standardised effect sizes in units of SD were estimated by fitting the same statistical models described above with the standardised scores of loneliness and wellbeing as the outcomes.

### Health economic analysis

#### Administrative cost of running a KbP exchange

To evaluate the costs of running a KbP exchange, we calculated both the total cost to the MHC of administering the exchange and the mean cost per exchange participant, by exchange phase. The amount of time spent on organising a KbP exchange was multiplied by the average cost per hour of the team members working on the exchange (salary and overheads). The total cost of running the exchange was then divided by the number of participants who took part, regardless of whether they had joined the present study. Other costs to the MHC that did not form part of the timesheets and were not included in this calculation are considered in the Discussion section. Although participants were expected to spend some money on materials and postage for the exchange of cards and letters, we did not collect information on participants’ out-of-pocket costs as this is not relevant to the NHS + PSS cost perspective chosen and would have burdened participants unnecessarily.

#### Healthcare resource use

Resource use was considered from the standard NHS health and personal social services cost perspective when designing the questionnaires [39]. The data capture focused on primary/community care as this was considered to be most relevant to the mechanism of KbP and collecting detailed information on additional service use was considered unnecessarily burdensome to participants and may have increased attrition. This was a relatively well population without any specific health conditions, so focusing on any type of disease-specific resource use was not considered to be appropriate. Nevertheless, we included a free text option to allow respondents to provide information on other health and social care service use, with a view to including some further questions in a future larger study if there were common responses there.

The feasibility of collecting health service resource use information with a view to future calculation of costs in a future cost-effectiveness analysis was assessed. We adapted the CSRI by using multiple choice answers in order to create a shorter and easier-to-complete CSRI to reduce participant burden. The questionnaire was multiple choice with pre-defined categories (i.e. 0, 1–2, 3–5 and 6 + contacts with services), and we report the number and proportion of participants for each category. We have broken down resource use by type of contact, exchange and time point. We planned to collect service use at baseline and 3 months only, with a reduced questionnaire excluding the CSRI being sent 2 weeks post-exchange. However, the same questionnaire was sent out at 2 weeks post-exchange and 3 months (Additional files 2 and 3). This resulted in an overlap in service use collected at baseline and 2 weeks post-exchange and therefore we only report service use and costs for baseline and 3 months follow-up.

#### Capability

Economic evaluations consider both costs and outcomes, and require a suitable outcome measure as well as cost information to provide a cost-effectiveness result. Most health-related analyses focus on health-related quality of life, and the EQ-5D-3L or 5L questionnaire is often the preferred tool for calculating quality-adjusted life-years (QALYs) [40]. As this is not an intervention aimed at a particular health concern, and is instead intended to improve wellbeing and reduce loneliness in a generally well population, a measure with a focus on broader wellbeing was considered more appropriate. The ICECAP-A is a’capability’ measure, which assesses an individual’s ability to engage in a set of’functionings ‘ or activities which contribute to wellbeing [41]. These include the level of attachment, stability, achievement, enjoyment and autonomy respondents feel able to achieve or enjoy [33]. This enables the calculation of a capability score using a standard tariff. The feasibility of collecting and calculating capability scores in this setting using the UK tariff [42] was assessed. Mean (SD) capability score is reported by time point for each exchange and both exchanges combined. Formal comparisons however were not made as the study was not powered to detect changes as it was a feasibility study.

### Attrition

An additional analysis investigated whether there were differences in terms of outcomes at baseline for those who answered follow-up questionnaires compared to those who did not. The analysis compared mean (SD) healthcare resource use cost and capability, wellbeing and loneliness scores for those who answered the post-exchange questionnaire compared to those who did not and the same for those who answered the 3-month follow-up questionnaire compared to those who did not. Two-tailed t-tests were used to investigate if any differences were statistically significant. The analysis was conducted separately for each exchange.

## Results

**Fig. 1:**
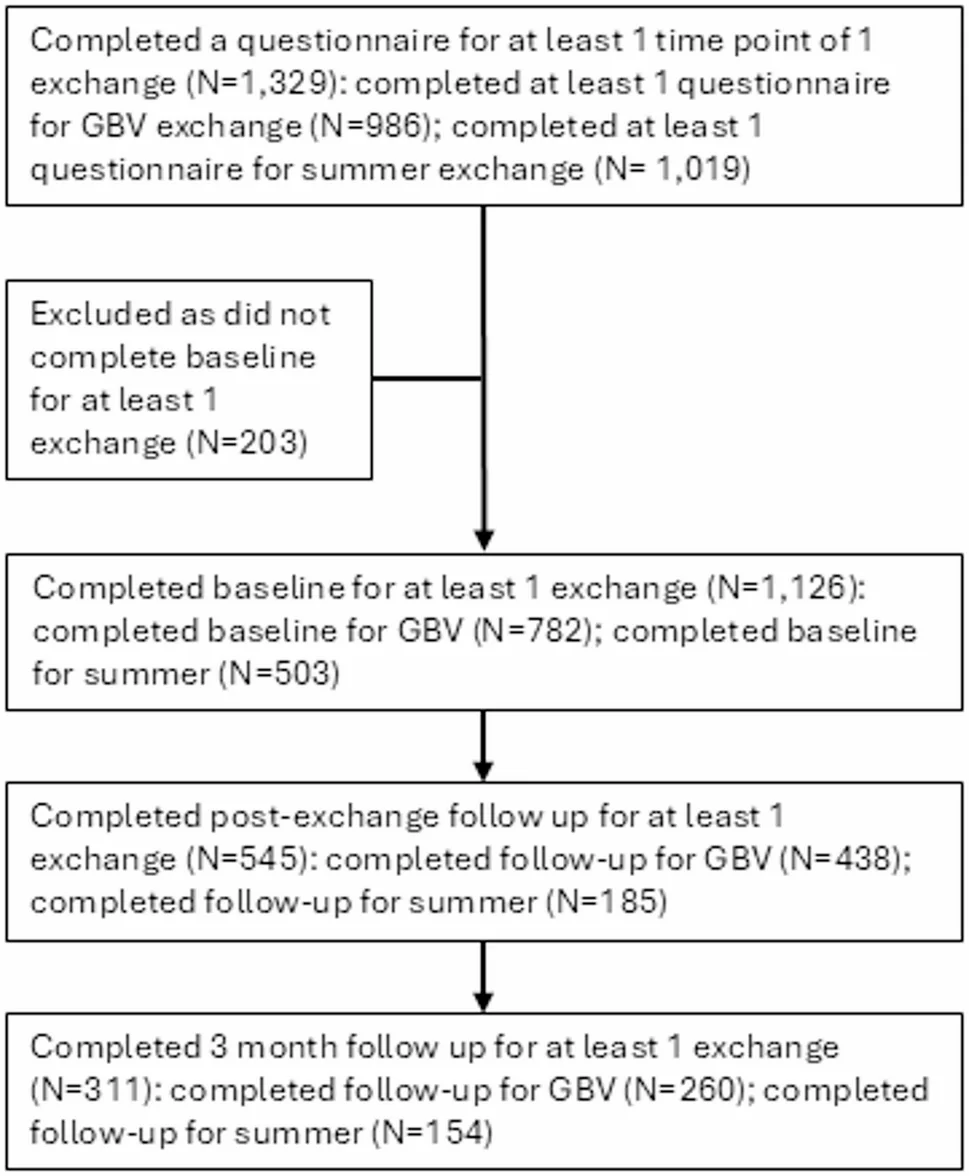
Participation throughout the KbP exchanges

Figure 1 shows the flow of participants through the two included KbP exchanges. Across the two exchanges, there were 1,285 responses from 1,142 unique participants, with 143 participants completing questionnaires for both exchanges. Retention rates were low, with only 438/782 (56%) of participants completing the post-exchange questionnaire for the GBV and only 185/503 (37%) for the summer exchange. For the 3-month questionnaire, 260/782 (33%) took part in follow-up for the GBV and 154/503 (31%) for the summer exchange.

Participant demographics are presented in Table 1. The age distribution of participants peaked at the 41 to 50 years age group (*n* = 296, 26%), with more than two-thirds of participants aged between 31 and 60 years of age (*n* = 789, 70%). The vast majority of participants were female (*n* = 1,075, 94%) and of white ethnicity (*n* = 1,078, 95%).

**Table 1:** Demographic information

	**Distinct individuals completing baseline surveys (*N* = 1,142)** n (%)
Age group	
Under 18 years	23 (2%)
18 to 30 years	186 (16%)
31 to 40 years	270 (24%)
41 to 50 years	296 (26%)
51 to 60 years	223 (20%)
Over 60 years	134 (12%)
Prefer not to say	10 (1%)
Gender	
Female	1,075 (94%)
Male	51 (4%)
Trans or non-binary	8 (1%)
Prefer not to say	8 (1%)
Ethnicity	
White	1,078 (95%)
Asian	12 (1%)
Black	9 (1%)
Mixed	22 (2%)
Other	8 (1%)
Prefer not to say	13 (1%)

Statistics are counts (n) and percentages (%) unless otherwise specified. Percentages are reported to the nearest whole number. There were missing demographics data for two participants (in addition to Prefer not to say responses)

### Wellbeing and loneliness

Table 2 presents the results of the analysis. Additional information can be found in Additional file 4. When data from both exchanges were combined, both loneliness and wellbeing improved over time, and these improvements were greater at 3 months than at 2 weeks post-exchange. Compared to baseline, loneliness was lower (i.e. people were less lonely) at the end of the KbP exchange (change in loneliness score = ‑0.21 units; 95% CI: −0.29 to −0.12) and at 3 months follow-up (change in loneliness score = −0.40; 95% CI: −0.50 to −0.30); wellbeing was greater immediately after the KbP exchange (change in wellbeing score = 0.68; 95% CI: 0.47 to 0.88) and at 3 months follow-up (change in wellbeing score = 1.01; 95% CI: 0.77 to 1.25).

**Table 2:** Estimated change in loneliness and wellbeing 2 weeks and 3 months after the Kindness by Post exchanges

	**Mean **(SD)	**Effect sizes on original scale**		**Standardised effect sizes**	
		**Estimated change in scale units **(95% CI)	***P* value**	**Estimated change in standard deviations **(95% CI)	***P* value**
Loneliness					
Baseline (*N* = 1,285)	5.98 (1.78)	–	–	–	–
2 weeks post exchange (*N* = 623)	5.73 (1.87)	−0.21 (−0.29 to −0.12)	< 0.001	−0.11 (−0.16 to −0.06)	< 0.001
3 months follow-up (*N* = 414)	5.57 (1.83)	−0.40 (−0.50 to −0.30)	< 0.001	−0.22 (−0.27 to −0.16)	< 0.001
Wellbeing					
Baseline (*N* = 1,285)	19.96 (3.48)	–	–	–	–
2 weeks post exchange (*N* = 623)	20.70 (3.92)	0.68 (0.47 to 0.88)	< 0.001	0.19 (0.13 to 0.26)	< 0.001
3 months follow-up (*N* = 414)	21.09 (3.71)	1.01 (0.77 to 1.25)	< 0.001	0.29 (0.22 to 0.36)	< 0.001

Summary statistics are mean (SD) unless otherwise specified. SD = standard deviation. CI = confidence interval. Counts after time points (n =) are number of observations, not number of individuals. Estimates of change were obtained from a multilevel model with a repeated measures outcome and a random effect of participant to account for correlations between repeated measures within individuals. To generate standardised effect sizes, scores at all three time points were standardised by the mean and SD of the measure at baseline

### Health economics

#### Administrative costs

Table 3 shows the different activities associated with each part of the exchange which were used as a basis for costing the exchanges’ administration. The costs of running KbP based on the quantity of time spent on each part of the exchange are presented in Table 4. A total of 39 h 5 min and 36 h 50 min were spent on the GBV and Summer exchanges, respectively. This led to a cost of £1655 for the GBV and £1186 for the Summer exchange. The time spent on the exchange does not seem to not increase substantially when the number of participants increases. The GBV had 469 more participants than the Summer exchange and a unit cost of £0.67 per participant compared with £0.87 for the Summer exchange.

**Table 3:** The phases of running KbP and associated activities

	Pre-registration	Sign up	Card sending phase	Back-up System	Close
Management	• Team meetings • Miscellaneous tasks				• Review exchange instructions for participants • Wrap-up meeting
Platform	• Updating website • Update timetable • Update FAQs		• Close registration • Trigger matches	• Check website functionality	• Delete cancellation data ahead of next exchange
Comms	• Prepare social media for partners • Prepare email for partners • Prepare press release	• Outreach to partners • Send press release • Respond to media requests • Ask past participants to help promote social media posts	• Repost social media		
SendInBlue email solution	• Update email template for exchange	• Send ‘nudge’ to unread registration emails	• Send reminder and donation emails	• Send back-up system emails to participants	• Send ‘bonnie in check’ email
Tech support (for participants)		• Resend confirmation links • Answer sign up queries • Remove users who have withdrawn,	• Process incorrect postal addresses • Process cancellations • Address queries from users	• Answer miscellaneous queries	• Answer miscellaneous queries

**Table 4:** Amount and cost of time spent on each exchange by the MHC including all routine task changes

	**GBV**				**SUMMER**			
	Director		Communications Manager		Director		Communications Manager	
	Time (minutes	Cost (£)	Time (minutes)	Cost (£)	Time (minutes	Cost (£)	Time (minutes	Cost (£)
Phase of exchange								
Pre-registration	390	£218.40	170	£66.30	310	£173.60	190	£74.10
Sign up	160	£89.60	75	£29.25	135	£75.60	75	£29.25
Card sending phase	335	£187.60	940	£366.60	290	£162.40	930	£362.70
Back-up system	120	£67.20	0	-	100	£56.00	0	-
Close	125	£70.00	30	£11.70	150	£84.00	30	£11.70
Total time (hh:mm) & cost (£)	18:50	£632.80	20:15	£473.85	16:25	£551.60	20:25	£477.75
Total cost per exchange	£1,106.65				£1,029.35			
Number of participants	1655*				1186*			
Cost per participant	*£*0.67				£0.87			

^*^Total number of participants in the exchange regardless of whether they also joined this study

There are other resources which contribute to the running of KbP, however many of these are offered pro bono and would be difficult to quantify (examples are presented in Additional file 5). Whilst many of these are unquantifiable and are mostly provided for “free”, it is important to acknowledge the donated time which goes into the exchanges and consider how some additional funding to areas such as advertising may enable the scaling up of the exchanges and allow more people to benefit from the potential improvements in loneliness and wellbeing.

#### Healthcare resource use

Table 5 reports the mean levels of resource use by service at each time point for both exchanges combined (resource use for each exchange are in Additional file 6; a summary of free text responses reporting other changes in health care is in Additional File 9).

**Table 5:** Healthcare Resource Use for both exchanges combined

Both exchanges combined	N (%)	
Number of times the service was accessed over preceding 3 months	**Baseline (*N* = 1285)**	**3 months **(*N* = 414)
General Practitioner		
0	635 (49%)	207 (50%)
1 - 2	501 (39%)	159 (38%)
3 - 5	130 (10%)	43 (10%)
6 +	19 (1%)	5 (1%)
Practice Nurse		
0	910 (71%)	277 (67%)
1 - 2	342 (27%)	122 (29%)
3 - 5	27 (2%)	10 (2%)
6 +	6 (0%)	5 (1%)
Speech & Language therapist, Occupational therapist, or Physiotherapist		
0	1178 (92%)	365 (88%)
1 - 2	66 (5%)	36 (9%)
3 - 5	30 (2%)	11 (3%)
6 +	11 (1%)	2 (0%)
NHS Direct/calls to 111		
0	1109 (86%)	360 (87%)
1 - 2	159 (12%)	47 (11%)
3 - 5	13 (0%)	5 (1%)
6 +	4 (0%)	2 (0%)
One-to-one talking therapy		
None	1048 (82%)	343 (83%)
Less than once a month	65 (5%)	20 (5%)
Less than once a week	50 (4%)	17 (4%)
Once a week	117 (9%)	32 (8%)
More than once a week	5 (0%)	2 (0%)
Group therapy		
None	1237 (96%)	401 (97%)
Less than once a month	24 (2%)	5 (1%)
Less than once a week	9 (1%)	7 (2%)
Once a week	10 (1%)	1 (0%)
More than once a week	5 (0%)	(0%)

#### Capability

Descriptive statistics for capability scores for both exchanges combined are shown in Table 6, and a breakdown for each exchange separately can be found in Additional file 7. Capability scores were calculated at baseline (mean (SD) = 0.74 (0.18)), post-exchange (0.75 (0.19)) and at 3-month follow-up (0.76 (0.18)).

**Table 6:** Mean capability scores by timepoint for both exchanges combined

	N	Mean (SD)
Both combined		
Baseline	1285	0.74 (0.18)
2 weeks post-exchange	831	0.75 (0.19)
3 months	425	0.76 (0.18)

### Attrition

There was a high drop-out rate, as seen in Table 6. There were no statistically significant differences in terms of costs, capability, wellbeing or loneliness scores at baseline for those who completed the follow-up questionnaires compared with those who did not. Additional file 8 reports demographics, mean (SD) baseline costs, capability, wellbeing and loneliness scores for those who completed follow-up questionnaires and those who did not.

## Discussion

### Main findings

We found some evidence that people’s wellbeing and loneliness had improved by the end of a KbP exchange and that these gains were sustained three months later. Since this was an observational, pre-post study, with high drop-out rates, causal attribution of improvements from participation in an exchange is unwarranted. However, our results were consistent with findings from an earlier evaluation of a KbP exchange [28], which found post-exchange gains in wellbeing and loneliness for a smaller sample. Taken together, we believe that there is preliminary and consistent evidence of a sustained reduction in feelings of loneliness and increase in wellbeing amongst participants during a KbP exchange. This is consistent with evidence from other studies, which has typically shown small-medium effects for random acts of kindness interventions targeting loneliness, such as The KIND Challenge, in which participants perform acts of kindness of their choosing in their communities [43]. We used a convenience sample of exchange participants, which was predominantly White and female, and our results may lack generalisability to other demographic groups.

Our cost analysis indicates the administrative cost of delivering the intervention to be around £0.67-£0.87 per participant, although it is worth noting that this estimate does not include time which individuals and other organisations have contributed pro-bono, which are thought to be considerable. The cheap cost of delivery, and its highly scalable design (similar cost when there were more people), suggest that KbP could be an effective and cost-effective large-scale intervention for improving public health, which we believe justifies future research to explore this possibility in a more definite manner.

Our results also demonstrate the feasibility of collecting the necessary clinical and health economic measures for a full effectiveness and cost-effectiveness analysis. Being mindful that overburdensome data collection was likely to be a significant deterrent to study participation, we chose to adapt the CSRI. While shortening the CSRI and swapping questions with multiple-choice items resulted in some loss of information due to wider categories and imputing the 6 + contacts category as 6 contacts, it resulted in a much shorter and easier-to-complete CSRI. We observed that only between 0 and 2% of respondents reported using the highest category of contacts (6 +), so anticipate that this loss of precision should not have a great impact on the results in future. The free text variables provided information suggesting that although this is a relatively well population, we should include use of community and secondary care mental health services and use of medications for common mental health conditions in future, as use of these was reported by up to around 10% of respondents. The other measures we included were chosen in part for their brevity. The brief nature of our study questionnaire allowed us to make all items compulsory, largely removing concerns related to incompleteness. However, we are unsure whether there were any questionnaire items that participants may have disliked and that could have contributed to the high drop-out rate. Anecdotal feedback from the MHC executive suggested that people found some of the ICECAP-A items “strange” in this context and based on the benefits patients reported in our realist evaluation [44], it may lack responsiveness to benefits from the exchange. In a future study the loss of information from using multiple choice questions in the CSRI would most likely be preferable to incomplete questionnaires or a low follow-up due to an overly burdensome version of the CSRI. However, questions about the suitability of the ICECAP-A to detect important improvements resulting from KbP as well as its acceptability to study participants suggest that it may be advisable to collect a different measure. A future study would instead use the EQ-5D-5L, which is a generic health-related quality of life instrument and likely to be more appropriate in this context.

### Strengths

This study is the largest evaluation to date of the KbP initiative, and our results indicate that it could potentially be an effective and cheap population health intervention for loneliness and mental wellbeing, which is scalable and relatively easy to deliver. This study builds upon the previous evaluations conducted by our research team [28] in a number of ways. We have improved the generalisability of findings by obtaining data from two KbP exchanges. It is also the first estimate of unit cost per participant of administering an exchange and has demonstrated the feasibility of conducting a health economic evaluation in future work. Moreover, it has allowed us to further refine our study procedures and helped us to identify appropriate domains/outcomes for measuring impact.

### Limitations

There are a number of methodological limitations of this study. Most importantly, we did not include a comparison group, making it impossible to causally attribute benefits observed at follow-up to the intervention. Participants’ mean wellbeing score at baseline was below the population norm, and so the observed improvement could be explained by regression to the mean. Secondly, there were high drop-out rates between follow-up data collections, allowing for the possibility that those providing outcome data at exchange end and 3 months post-exchange are systematically different from those who did not complete the follow-up questionnaires. While we found no evidence of baseline differences between completers and drop-outs in outcome data, it is possible that those who did not feel that they derived any benefit from KbP were less inclined to remain in the study potentially inflating our effect estimates. Thirdly, given the design of the questionnaire it was difficult to tell if the low retention rate was due to a question or group of questions in particular or whether the option to not answer all questions would have led to a higher response rate. Fourthly, we used the ICECAP-A, but capability may not have been the domain impacted most by the intervention. It may be that a different health economic outcome measure is preferable. Equally, we explored the possibility of measuring service use, which seemed feasible, and in order to understand and measure the true impact of the intervention, while being appropriately targeted and minimising burden on study participants, we used a reduced version of the CSRI, specially modified for this study. However, it may be that our changes have reduced the sensitivity of the CSRI to service use and so we may not be able to correctly estimate the broader impact of the intervention in this regard. Fifthly, we did not consider the out-of-pocket costs to participants in the exchanges, such as art materials and postage, as these were not relevant to the operational costs of running a KbP from the perspective of the MHC. However, these costs could represent a barrier to participation and so would be a valuable addition to a future evaluation. Sixthly, while we recorded which exchanges each participant took part in during the study period, we did not collect data on which and how many exchanges participants took part in before our study. This prevents us from exploring longer term trends between wellbeing and loneliness and the number of exchanges someone has participated in during their lifetimes. Seventhly, the sample included a high proportion of white and female participants, so findings may not apply to a more diverse population. As discussed in our realist evaluation, a potential explanation for this lack of diversity is that KbP may have low appeal to some communities/groups because it relies on postal cards [44]. Other ways of expressing kindness, such as gifting food or offers of help in the home, may be preferred by some groups. Finally, we did not collect data on lifestyle or other changes that participants may have made during the study period, which is a potential source of confounding.

### Implications for practice

This study was conducted in the context of the pandemic, a period of significantly heightened social isolation for many. Data collected during the pandemic by the Office for National Statistics (ONS) from March 2020 to March 2022 [3], and the Understanding Society COVID-19 Study, a special wave of the UK Household Longitudinal Study (UKHLS) [45] conducted in April 2020, showed that between 30–40% of adults in the UK felt lonely at least some of the time and that their well-being had been affected by their feeling lonely in the past 7 days. This was highest amongst women (42%; compared to 27% of men) [32]. Across both KbP exchanges, 94% of participants were female and 95% of white ethnicity. Notably, the mean wellbeing score at baseline for our KbP participants of 19.96 is almost a full standard deviation below the population norm (23.59 for women [46]). This suggests KbP is reaching people with low wellbeing, and therefore at higher risk of depression or other mental health conditions. This is a vulnerable and underserved population, and so a public mental health programme such as KbP could be a useful intervention to support this group and perhaps help improve the social inequalities that exist in relation to mental health [47]. As such, KbP is well targeted to a population with notably high levels of loneliness and social isolation. Our results provide an indication that KbP may be effective in reducing loneliness and improving wellbeing in this population, which is consistent with the wider literature on the health benefits of random acts of kindness. Even though the estimated effect size is likely to be small, as it is cheap and potentially highly scalable, KbP represents a promising, participatory public mental health initiative that could be commissioned or provided at scale to try to improve public wellbeing and address loneliness. Since it is an intervention that people can engage with from their home, it has the potential to engage and benefit people vulnerable to experiencing social isolation and loneliness. However, it is important to note that the fact that KbP has not reached an ethnically diverse population or many men, representative of the general population, limits its current potential as a public mental health intervention.

### Implications for research

The results of this evaluation, taken alongside our previous research [28] provide a promising signal of potential efficacy of KbP as a public health intervention for loneliness and wellbeing. We have also provided an initial estimate of the cost per participant of running a KbP exchange, but with the noted caveats that these costs could reduce should the initiative be scaled up or increase if the services that the organisers rely on be no longer provided without cost. We have also demonstrated the feasibility of collecting the health and economic measures needed for a full effectiveness and cost-effectiveness analysis. In parallel to the present study, we have also conducted a process evaluation that provides us with a logic model of the mechanism of action of the intervention [44]. Some of the key findings include that participants spoke positively about both giving and receiving post, as well as the sense of community associated with KbP and the feeling that they are helping others. Connection with a stranger, rather than friends or family, was also an important feature. Participants spoke about these benefits being sustained over time through keeping the cards and by engaging with the social media communities related to KbP exchanges. Some also spoke about how these benefits build over time as they participate in more exchanges. Taken together, this work sets the groundwork for a future, definitive study, such as a randomised controlled trial.

We believe that there are a number of important considerations for any such future research. Ideally a robust evaluation of KbP cost-effectiveness should be included, considering the perspective of the body or organisation which would bear the costs of providing the service as well as costs for participants, and options for how best to measure this should be considered. Quality-adjusted life-years (QALYs) calculated from responses to the EQ-5D-5L may be preferable to Years of Full Capability (YFCs) calculated from responses to the ICECAP-A, and this would also bring the outcome measure in line with the NICE Reference Case for economic evaluations [35]. We believe that it would also be valuable to explore the reach or appeal of KbP to different communities or groups. While our process evaluation helps to understand the potential barriers and facilitators to participation, further research is needed with currently under-represented groups. Increasing engagement in KbP-type Acts of Kindness interventions may involve taking more targeted approaches while keeping the current KbP exchange design, or it could involve alternative exchange designs that are more acceptable to specific groups/communities. Part of this could include identifying strategies for improving the visibility or acceptability of such initiatives to diverse populations, and it would be valuable to do this either before or as a component of future research. Although, if novel exchange designs are developed, while the findings of the present study may offer promising evidence of the potential benefits, further research would need be needed to confirm the effectiveness of those alternative designs.

## Supplementary Information

Additional file 1.

Additional file 2.

Additional file 3.

Additional file 4.

Additional file 5.

Additional file 6.

Additional file 7.

Additional file 8.

Additional file 9.

## Data Availability

The datasets used and/or analysed during this study are available from the corresponding author on reasonable request.

## Abbreviations

KbP: Kindness by Post
MHC: Mental Health Collective
GBV: Great British Valentine
SWEMWBS: Short Warwick-Edinburgh Mental Wellbeing Scale
ULS-3: University of California at Los Angeles Loneliness Scale
ICECAP-A: ICEpop CAPability measure for Adults
CSRI: Client Service Receipt Inventory
SD: Standard Deviation
PSSRU: Personal Social Services Research Unit
ONS: Office for National Statistics
YFC: Years of Full Capability
