# Supplementary material for: #KindnessbyPost: a pre-post, observational evaluation and feasibility study of a participatory public mental health intervention
Source: BMC Public Health. 2026 Jul 22;26:2997. doi: 10.1186/s12889-026-28170-x (PMC13679209; doi:10.1186/s12889-026-28170-x)
Supplement: Supplementary file 2 [file 12889_2026_28170_MOESM2_ESM.pdf]

Summer #KindnessByPost: "After" Questionnaire

All your feedback will be kept completely anonymous and analysed by independent researchers. We use your email address for administrative purposes only.

doej66374@gmail.com Switch accounts

*Required

Email *

Your email address

How many times have you taken part in #KindnessByPost? *

- ◯ This was my first time
- ◯ I've taken part once before
- ◯ I've taken part twice before
- ◯ I've taken part at least three times before

Did you send a card or letter for #KindnessByPost? *

- ◯ Yes
- ◯ No

Did you receive something for #KindnessByPost yourself? *

- ◯ Yes, I got a card in the first round
- ◯ Yes, I got a back-up card
- ◯ No

Tell us any comments about your experience of taking part in the project

Your answer

Question 1. *

	Hardly ever or never	Some of the time	Often
How often do you feel you lack companionship?	◯	◯	◯
How often do you feel left out?	◯	◯	◯
How often do you feel isolated from others?	◯	◯	◯

Question 2. *

Below are some statements about feelings and thoughts. Please tick the box that best describes your experience of the last two weeks.

	None of the time	Rarely	Some of the time	Often	All of the time
"I've been feeling optimistic about the future"	○	○	○	○	○
"I've been feeling useful"	○	○	○	○	○
"I've been feeling relaxed"	○	○	○	○	○
"I've been dealing with problems well"	○	○	○	○	○
"I've been thinking clearly"	○	○	○	○	○
"I've been feeling close to other people"	○	○	○	○	○
"I've been able to make up my own mind about things"	○	○	○	○	○

Question 3 *

Feeling settled and secure

- ◯ I am able to feel settled and secure in **all** areas of my life
- ◯ I am able to feel settled and secure in **many** areas of my life
- ◯ I am able to feel settled and secure in **a few** areas of my life
- ◯ I am **unable** to feel settled and secure in **any** areas of my life

Question 4 *

Love, friendship and support

- ◯ I can have **a lot** of love, friendship and support
- ◯ I can have **quite a lot** of love, friendship and support
- ◯ I can have **a little** love, friendship and support
- ◯ I **cannot** have **any** love, friendship and support

Question 5. *

Being independent

- ◯ I am able to be **completely** independent
- ◯ I am able to be independent in **many** things
- ◯ I am able to be independent in **a few** things
- ◯ I am **unable** to be at all independent

Question 6. *

Achievement and progress

- I can achieve and progress in **all** aspects of my life
- I can achieve and progress in **many** aspects of my life
- I can achieve and progress in **a few** aspects of my life
- I **cannot** achieve and progress in **any** aspects of my life

Question 7. *

Enjoyment and pleasure

- I can have **a lot** of enjoyment and pleasure
- I can have **quite a lot** of enjoyment and pleasure
- I can have **a little** enjoyment and pleasure
- I **cannot** have **any** enjoyment and pleasure

Question 8. *

How many times have you accessed these healthcare services over the past 3 months? (**mobile users** - scroll right for more options)

	0 times	1-2 times	3-5 times	6+ times
General Practitioner (GP)	○	○	○	○
Practice Nurse (GP Nurse)	○	○	○	○
Speech and Language Therapist, Occupational Therapist, or Physiotherapist	○	○	○	○
NHS direct or Call 111	○	○	○	○

Question 9. *

Have you had any talking therapy sessions (e.g. psychotherapy, counselling or other therapy at an Improving Access to Psychological Therapies (IAPT) service – NHS, Private, or non-profit) in the last 3 months?

	0 times	less than once a month	less than once a week	once per week	More than once a week
One-to-one (individual)	○	○	○	○	○
Group	○	○	○	○	○

Question 10

Have there been any other changes to the healthcare support you have accessed over the last 3 months? Please give brief details.

Your answer

Send me a copy of my responses.

Submit

Clear form

Never submit passwords through Google Forms.

reCAPTCHA
Privacy Terms

This content is neither created nor endorsed by Google. Report Abuse - Terms of Service - Privacy Policy

Google Forms
