# Supplementary material for: #KindnessbyPost: a pre-post, observational evaluation and feasibility study of a participatory public mental health intervention
Source: BMC Public Health. 2026 Jul 22;26:2997. doi: 10.1186/s12889-026-28170-x (PMC13679209; doi:10.1186/s12889-026-28170-x)
Supplement: Supplementary file 4 [file 12889_2026_28170_MOESM4_ESM.docx]

# Additional File 6: Additional results from the analysis of loneliness and wellbeing outcomes

*Table 1: Estimated change in loneliness 2 weeks and 3 months after the Kindness By Post exchanges*

|  | **Mean**  **(SD)** | **Effect sizes on original scale** | | **Standardised effect sizes** | |
| --- | --- | --- | --- | --- | --- |
|  |  | **Estimated change in scale units**  **(95% CI)** | **P value** | **Estimated change in standard deviations** **(95% CI)** | **P value** |
|  | | | | | |
| Baseline  (N = 1,285) | 5.98  (1.78) | – | – | – | – |
| 2 weeks post exchange  (N = 623) | 5.73  (1.87) | Fixed effects: -0.21  (-0.29 to -0.12) | <0.001 | Fixed effects: -0.11  (-0.16 to -0.06) | <0.001 |
| 3 months follow-up (N = 414) | 5.57  (1.83) | -0.40  (-0.50 to -0.30) | <0.001 | -0.22  (-0.27 to -0.16) | <0.001 |
|  |  | Random effects: 2.69 (2.39 to 3.03) |  | Random effects: 0.85 (0.75 to 0.96) |  |
|  |  | Likelihood Ratio test = 1389.07 | <0.001 | Likelihood Ratio test = 1333.21 | <0.001 |

*Table 2: Estimated change in wellbeing 2 weeks and 3 months after the Kindness By Post exchanges*

|  | **Mean**  **(SD)** | **Effect sizes on original scale** | | **Standardised effect sizes** | |
| --- | --- | --- | --- | --- | --- |
|  |  | **Estimated change in scale units**  **(95% CI)** | **P value** | **Estimated change in standard deviations** **(95% CI)** | **P value** |
|  | | | | | |

| Baseline  (N = 1,285) | 19.96 (3.48) | – | – | – | – |
| --- | --- | --- | --- | --- | --- |
| 2 weeks post exchange  (N = 623) | 20.70 (3.92) | Fixed effects: 0.68  (0.47 to 0.88) | <0.001 | Fixed effects: 0.19  (0.13 to 0.26) | <0.001 |
| 3 months follow-up  (N = 414) | 21.09 (3.71) | 1.01  (0.77 to 1.25) | <0.001 | 0.29  (0.22 to 0.36) | <0.001 |
|  |  | Random effects: 9.54 (8.41 to 10.83) |  | Random effects: 0.80 (0.71 to 0.91) |  |
|  |  | Likelihood Ratio test = 977.03 | <0.001 | Likelihood Ratio test = 987.81 | <0.001 |

*Summary statistics are mean (SD) unless otherwise specified. SD = standard deviation. CI = confidence interval. Counts after time points (n = ) are number of observations, not number of individuals. Estimates of change were obtained from a multilevel model with a repeated measures outcome and a random effect of participant to account for correlations between repeated measures within individuals. To generate standardised effect sizes, scores at all three time points were standardised by the mean and SD of the measure at baseline.*
