# Supplementary material for: #KindnessbyPost: a pre-post, observational evaluation and feasibility study of a participatory public mental health intervention
Source: BMC Public Health. 2026 Jul 22;26:2997. doi: 10.1186/s12889-026-28170-x (PMC13679209; doi:10.1186/s12889-026-28170-x)
Supplement: Supplementary file 5 [file 12889_2026_28170_MOESM5_ESM.docx]

# Additional File 7: Examples of services and resources provided to the Mental Health Collective for free

- IT support is provided pro bono by MHC’s IT partner, on an ad hoc basis. Much of the support provided so far has been provided during the initial set of KbP which would be considered a sunk cost and so it would be difficult to quantify how much the cost is expected to be per exchange at this stage. The IT partner have reported they consider to be remunerated by the improvements in wellbeing employees report from doing something “good” with their employed time.
- Hiring costs for the MHC.
- Emotional support provided by other employees during the course of the exchange.
- Liaising with volunteers, donors and partners.
- Advertising – currently mostly done through social media
