# Supplementary material for: #KindnessbyPost: a pre-post, observational evaluation and feasibility study of a participatory public mental health intervention
Source: BMC Public Health. 2026 Jul 22;26:2997. doi: 10.1186/s12889-026-28170-x (PMC13679209; doi:10.1186/s12889-026-28170-x)
Supplement: Supplementary file 6 [file 12889_2026_28170_MOESM6_ESM.docx]

# Additional File 9: Healthcare Resource use by exchange and combined

| **Great British Valentine** | **N (%)** | |
| --- | --- | --- |
| Number of times the service was accessed over 3 months | **Baseline**  (N=782) | **3 months**  (N=260) |
| **General Practitioner** | | |
| 0 | 397 (51%) | 139 (53%) |
| 1 - 2 | 301 (38%) | 92 (35%) |
| 3 - 5 | 76 (10%) | 25 (10%) |
| 6+ | 8 (1%) | 4 (2%) |
| **Practice Nurse** | | |
| 0 | 560 (72%) | 177 (68%) |
| 1 - 2 | 204 (26%) | 71 (27%) |
| 3 - 5 | 15 (2%) | 8 (3%) |
| 6+ | 3 (0%) | 4 (2%) |
| **Speech & Language therapist, Occupational therapist, or Physiotherapist** | | |
| 0 | 722 (92%) | 229 (88%) |
| 1 - 2 | 40 (5%) | 25 (10%) |
| 3 - 5 | 14 (2%) | 5 (2%) |
| 6+ | 6 (1%) | 1 (0%) |
| **NHS Direct/calls to 111** | | |
| 0 | 685 (88%) | 229 (88%) |
| 1 - 2 | 86 (11%) | 25 (10%) |
| 3 - 5 | 9 (0%) | 5 (2%) |
| 6+ | 2 (0%) | 1 (0%) |
| **One-to-one talking therapy** | | |
| None | 632 (81%) | 253 (97%) |
| less than once a month | 45 (6%) | 1 (0%) |
| less than once a week | 27 (3%) | 5 (2%) |
| once a week | 73 (9%) | 1 (0%) |
| more than once a week | 5 (1%) | 0 (0%) |
| **Group therapy** | | |
| None | 751 (96%) | 253 (97%) |
| less than once a month | 18 (2%) | 1 (0%) |
| less than once a week | 5 (1%) | 5 (2%) |
| once a week | 4 (1%) | 1 (0%) |
| more than once a week | 4 (1%) | 0 (0%) |
| **Summer Exchange** | **N (%)** | |
| Number of times the service was accessed over 3 months | **Baseline**  (N=503) | **3 months**  (N=154) |
| **General Practitioner** | | |
| 0 | 238 (47%) | 68 (44%) |
| 1 - 2 | 200 (40%) | 67 (44%) |
| 3 - 5 | 54 (11%) | 18 (12%) |
| 6+ | 11 (2%) | 1 (1%) |
| **Practice Nurse** | | |
| 0 | 350 (70%) | 100 (65%) |
| 1 - 2 | 138 (27%) | 51 (33%) |
| 3 - 5 | 12 (2%) | 2 (1%) |
| 6+ | 3 (1%) | 1 (1%) |
| **Speech & Language therapist, Occupational therapist, or Physiotherapist** | | |
| 0 | 456 (91%) | 128 (83%) |
| 1 - 2 | 26 (5%) | 23 (15%) |
| 3 - 5 | 16 (3%) | 3 (2%) |
| 6+ | 5 (1%) | 0 (0%) |
| **NHS Direct/calls to 111** | | |
| 0 | 424 (84%) | 131 (85%) |
| 1 - 2 | 73 (15%) | 22 (14%) |
| 3 - 5 | 4 (0%) | 1 (1%) |
| 6+ | 2 (0%) | 0 (0%) |
| **One-to-one talking therapy** | | |
| None | 416 (83%) | 131 (85%) |
| less than once a month | 20 (4%) | 8 (5%) |
| less than once a week | 23 (5%) | 6 (4%) |
| once a week | 44 (9%) | 8 (5%) |
| more than once a week | 0 (0%) | 1 (1%) |
| **Group therapy** | | |
| None | 486 (97%) | 148 (96%) |
| less than once a month | 6 (1%) | 4 (3%) |
| less than once a week | 4 (1%) | 2 (1%) |
| once a week | 6 (1%) | 0 (0%) |
| more than once a week | 1 (0%) | 0 (0%) |
