# Supplementary material for: #KindnessbyPost: a pre-post, observational evaluation and feasibility study of a participatory public mental health intervention
Source: BMC Public Health. 2026 Jul 22;26:2997. doi: 10.1186/s12889-026-28170-x (PMC13679209; doi:10.1186/s12889-026-28170-x)
Supplement: Supplementary file 7 [file 12889_2026_28170_MOESM7_ESM.docx]

# Additional File 10: Mean capability score by timepoint and exchange

Appendix 7: Mean capability scores by timepoint and exchange

| **GBV** |  |  |
| --- | --- | --- |
| Baseline | 782 | 0.74 (0.18) |
| Post Exchange | 639 | 0.76 (0.19) |
| 3 months | 264 | 0.78 (0.18) |
| **Summer** |  |  |
| Baseline | 503 | 0.74 (0.19) |
| Post Exchange | 192 | 0.73 (0.19) |
| 3 months | 161 | 0.74 (0.19) |
