# Supplementary material for: #KindnessbyPost: a pre-post, observational evaluation and feasibility study of a participatory public mental health intervention
Source: BMC Public Health. 2026 Jul 22;26:2997. doi: 10.1186/s12889-026-28170-x (PMC13679209; doi:10.1186/s12889-026-28170-x)
Supplement: Supplementary file 8 [file 12889_2026_28170_MOESM8_ESM.docx]

# Additional File 11: Attrition analysis

Table 1 – Demographics of participants followed up and lost to follow up

|  | **Followed up at least once (N= 876)** n(%) | **Lost to follow up (N=266)** n(%) | **Total (N = 1,142)**  n (%) |
| --- | --- | --- | --- |
| Age group  Under 18 years  18 to 30 years  31 to 40 years  41 to 50 years  51 to 60 years  Over 60 years  Prefer not to say | 17 (2%)  116 (13%)  200 (23%)  235 (27%)  185 (21%)  113 (13%)  10 (1%) | 6 (2%)  70 (26%)  70 (26%)  61 (23%)  38 (14%)  21 (8%)  0 (0%) | 23 (2%)  186 (16%)  270 (24%)  296 (26%)  223 (20%)  134 (12%)  10 (1%) |
| Gender  Female  Male  Trans or non-binary  Prefer not to say | 827 (94%)  34 (4%)  7 (1%)  8 (1%) | 248 (93%)  17 (6%)  1 (0%)  0 (0%) | 1,075 (94%)  51 (4%)  8 (1%)  8 (1%) |
| Ethnicity  White  Asian  Black  Mixed  Other  Prefer not to say | 830 (95%)  6 (1%)  7 (1%)  15 (2%)  5 (1%)  13 (1%) | 248 (93%)  6 (2%)  2 (1%)  7 (3%)  3 (1%)  0 (0%) | 1,078 (95%)  12 (1%)  9 (1%)  22 (2%)  8 (1%)  13 (1%) |

Table 2 – costs of participants followed up and lost to follow up

|  | **Post exchange** | | **3 months follow up** | |
| --- | --- | --- | --- | --- |
|  | Followed up | Missing Post-exchange | Followed up | Missing at 3 months |
| **GBV** |  |  |  |  |
| N (%) | 438 (56%) | 344 (44%) | 260 (33%) | 522 (67%) |
| Mean (SD) baseline cost | £173 (£319) | £180 (£335) | £185 (£342) | £172 (£318) |
| Mean (SD) baseline capability score | 0.51 (0.22) | 0.52 (0.21) | 0.50 (0.23) | 0.52 (0.21) |
| Mean (SD) baseline wellbeing score | 19.85 (3.53) | 19.65 (3.36) | 19.95 (3.75) | 19.67 (3.30) |
| Mean (SD) baseline loneliness score | 5.96 (1.80) | 6.07 (1.81) | 5.97 (1.80) | 6.03 (1.81) |
| **Summer** |  |  |  |  |
| N (%) | 185 (37%) | 318 (63%) | 154 (31%) | 349 (69%) |
| Mean (SD)baseline cost | £152 (£267) | £182 (£311) | £180 (£292) | £167 (£297) |
| Mean (SD) baseline capability score | 0.52 (0.22) | 0.52 (0.21) | 0.50 (0.22) | 0.53 (0.21) |
| Mean (SD) baseline wellbeing score | 20.67 (3.93) | 20.27 (3.29) | 20.47 (3.59) | 20.39 (3.53) |
| Mean (SD) baseline loneliness score | 5.80 (1.78) | 5.94 (1.70) | 5.79 (1.86) | 5.93 (1.67) |
