# Supplementary material for: #KindnessbyPost: a pre-post, observational evaluation and feasibility study of a participatory public mental health intervention
Source: BMC Public Health. 2026 Jul 22;26:2997. doi: 10.1186/s12889-026-28170-x (PMC13679209; doi:10.1186/s12889-026-28170-x)
Supplement: Supplementary file 9 [file 12889_2026_28170_MOESM9_ESM.docx]

# Additional File 11: Main mentions in 'Other changes in health care support' (free text)

In the free text box for question 10, asking, “Have there been any other changes to the health care support you have accessed over the last 3 months? Please give brief details.” The following categories reflect people’s responses. The majority said they had no health care needs; some mentioned the lack of available appointments, or lack of available in person appointments (i.e. appointments were only available by phone or online/video), due to Covid and general backlog of work. Some also specifically mentioned other mental health services they were accessing or were waiting to access, including counselling (sometimes it was specified whether this was through IAPT, Time to Talk, charities or private providers and sometimes it was not, so this detail has not been used to classify responses due to incomplete information potentially leading to bias); some reported use of social care or community mental health services, including crisis teams; some reported use of secondary care mental health services or A&E visits for mental health reasons; and some mentioned changing, increasing or decreasing doses of antidepressants, anxiety medications, or mood stabilizers. Some respondents reported health care used due to physical ailments such as diabetes or knee surgery, but these were classified as “none required” in the absence of any mention of mental health issues as they were considered not to be related to mental health, which is the focus of this intervention.

This information could be used in a future larger study to justify the inclusion of more detailed resource use questions in this relatively well population.

| **Main mentions in 'Other changes in health care support' (free text)** | | |
| --- | --- | --- |
| **Both exchanges combined** | **Total=** | **662** |
| **Before** | **n** | **%** |
| None required | 501 | 76% |
| Delay or lack of access | 71 | 11% |
| Online or video only | 11 | 2% |
| Phone | 23 | 3% |
| MH counselling/therapy | 30 | 5% |
| MH meditation/mindfulness | 4 | 1% |
| MH medications | 13 | 2% |
| MH social/community support | 1 | 0% |
| MH secondary care | 4 | 1% |
| MH A&E visit | 2 | 0% |
| **3 months** | **Total=** | **246** |
| None required | 191 | 78% |
| Delay or lack of access | 24 | 10% |
| Online or video only | 4 | 2% |
| Phone | 4 | 2% |
| MH counselling/therapy | 10 | 4% |
| MH meditation/mindfulness | 0 | 0% |
| MH medications | 6 | 2% |
| MH social/community support | 3 | 1% |
| MH secondary care | 4 | 2% |
| MH A&E visit | 0 | 0% |

MH = mental health, A&E = accident and emergency

| **Main mentions in 'Other changes in health care support' (free text)** | | |
| --- | --- | --- |
| **Great British Valentine** | **Total=** | **407** |
| **Before** | **n** | **%** |
| None required | 297 | 73% |
| Delay or lack of access | 45 | 11% |
| Online or video only | 7 | 2% |
| Phone | 17 | 4% |
| MH counselling/therapy | 20 | 5% |
| MH meditation/mindfulness | 4 | 1% |
| MH medications | 10 | 2% |
| MH social/community support | 1 | 0% |
| MH secondary care | 2 | 0% |
| MH A&E visit | 2 | 0% |
| **3 months** | **Total=** | **155** |
| None required | 118 | 76% |
| Delay or lack of access | 16 | 10% |
| Online or video only | 3 | 2% |
| Phone | 3 | 2% |
| MH counselling/therapy | 6 | 4% |
| MH meditation/mindfulness | 0 | 0% |
| MH medications | 5 | 3% |
| MH social/community support | 0 | 0% |
| MH secondary care | 4 | 3% |
| MH A&E visit | 0 | 0% |

MH = mental health, A&E = accident and emergency

| **Main mentions in 'Other changes in health care support' (free text)** | | |
| --- | --- | --- |
| **Summer exchange** | **Total=** | **255** |
| **Before** | **n** | **%** |
| None required | 204 | 80% |
| Delay or lack of access | 26 | 10% |
| Online or video only | 4 | 2% |
| Phone | 6 | 2% |
| MH counselling/therapy | 10 | 4% |
| MH meditation/mindfulness | 0 | 0% |
| MH medications | 3 | 1% |
| MH social/community support | 0 | 0% |
| MH secondary care | 2 | 1% |
| MH A&E visit | 0 | 0% |
| **3 months** | **Total=** | **91** |
| None required | 73 | 80% |
| Delay or lack of access | 8 | 9% |
| Online or video only | 1 | 1% |
| Phone | 1 | 1% |
| MH counselling/therapy | 4 | 4% |
| MH meditation/mindfulness | 0 | 0% |
| MH medications | 1 | 1% |
| MH social/community support | 3 | 3% |
| MH secondary care | 0 | 0% |
| MH A&E visit | 0 | 0% |

MH = mental health, A&E = accident and emergency
